# Circulating exosomal microRNAs as prognostic biomarkers for non-small-cell lung cancer

**DOI:** 10.18632/oncotarget.14369

**Published:** 2016-12-30

**Authors:** Qingyun Liu, Zubin Yu, Shuai Yuan, Weijia Xie, Chengying Li, Zeyao Hu, Ying Xiang, Na Wu, Long Wu, Li Bai, Yafei Li

**Affiliations:** ^1^ Department of Epidemiology, College of Preventive Medicine, Third Military Medical University, Chongqing, People's Republic of China; ^2^ Department of Thoracic Surgery, Xinqiao Hospital, Third Military Medical University, Chongqing, People's Republic of China; ^3^ Department of Respiratory Disease, Xinqiao Hospital, Third Military Medical University, Chongqing, People's Republic of China

**Keywords:** non-small-cell lung cancer (NSCLC), exosome, miRNAs, prognosis, biomarker

## Abstract

Exosomal miRNAs are proposed as excellent candidate biomarkers for clinical applications. However, little is known about their potential roles as prognostic biomarkers in lung cancer. In this study, we explored the prognostic value of plasma exosomal microRNAs (miRNAs) for non-small-cell lung cancer (NSCLC). Using a quantitative polymerase chain reaction (qPCR) array panel, we analyzed 84 plasma exosomal miRNAs in 10 lung adenocarcinoma patients and 10 matched healthy controls. The qPCR array showed 30 aberrantly expressed exosomal miRNAs. Nine candidate miRNAs were selected based on differential expression and previous reports for further evaluating their prognostic roles in 196 NSCLC patients. Elevated levels of exosomal miR-23b-3p, miR-10b-5p and miR-21-5p were independently associated with poor overall survival (with hazard ratio [95% confidence interval]: 2.42 (1.45 - 4.04), P = 0.001; 2.22 (1.18 - 4.16), P = 0.013; 2.12 (1.28 - 3.49), P = 0.003, respectively). When compared to the clinical prognostic variables only model, adding the three exosomal miRNA signatures significantly improved survival predictive accuracy with an increase of time-dependent area under the receiver operating characteristic curve from 0.88 to 0.91 (P=0.015). Our results indicated that plasma exosomal miR-23b-3p, miR-10b-5p and miR-21-5p are promising non-invasive prognostic biomarkers of NSCLC.

## INTRODUCTION

Lung cancer is one of the major causes of cancer-related deaths worldwide [1]. Non-small-cell lung cancer (NSCLC) accounts for about 80% of all cases of lung cancer. Although new therapies have improved the outcomes of patients with NSCLC, the predicted 5-year survival rate is only 15.9% [2]. Clinical factors, such as age, tumor stage, histological type and treatment modality are conventional predictors for the prognosis of NSCLC. However, blood-based biomarkers have become more and more appealing in NSCLC diagnosis and prognosis.

Exosomes are membrane-enclosed extracellular vesicles (EVs), with a diameter of 30-100nm [3]. Unlike microvesicles, another type of EV directly budding from plasma membrane, exosomes originate from endosomes [4]. During their maturing process, endosomes can accumulate intraluminal vesicles (ILVs), which are formed by inward budding of endosomal membrane. These endosomes then become multivesicular bodies (MVBs). Membrane of MVBs can fuse with plasma membrane, leading ILVs to be released as exosomes [4–6]. The production and release of exosomes is precisely regulated by several proteins, including YKT6, which is in turn regulated by miR-134 and miR-135b [7]. Almost all cell types can secrete exosomes under normal and stressful conditions, such as reticulocytes, dendritic cells, adipocytes, endothelial, and epithelial cells [6]. Cancer cells in particular are known to secrete more exosomes than normal cells of the same organ type [6]. The exosomal membrane is enriched in endosome-related membrane transport and fusion proteins (flotillin, annexins, GTPases), tetraspanin (CD9, CD63, CD81 and CD82), and MVB biogenesis-related proteins (Alix, TSG101) [8]. These enriched proteins are widely used as markers in exosome research.

Exosomes can be isolated from diverse body fluids, including plasma, serum, semen, urine, saliva, breast milk, amniotic fluid, ascites fluid, cerebrospinal fluid, and bile [4], and are very stable when stored at -20˚C [9]. Exosomes carry many kinds of biological molecules, including proteins, RNAs, DNAs and lipids [6, 10]. These molecules are protected by the lipid bilayer [9]. Recent publications have revealed diagnostic and prognostic roles of exosome for cancers [11, 12].

MicroRNAs (miRNAs) encapsulated in exosome are very stable [13, 14], and miRNA is the main component of exosomal small RNA [14]. Recent studies have indicated that exosomal miRNAs are excellent candidate biomarkers for clinical applications [15–18]. Huang *et al*. revealed that exosomal miR-1290 and miR-375 were promising prognostic biomarkers for castration-resistant prostate cancer [19]. Cazzoli *et al*. found a potential screening and diagnosing value of exosomal miRNAs for lung adenocarcinoma [13]. Taverna *et al*. and Giallombardo *et al*. reported that it was feasible to isolate and characterize exosomes in serum of NSCLC patients, and that exosomal miRNAs could be detected by qPCR [20, 21]. To the best of our knowledge, there has not been any report evaluating the prognostic role of exosomal miRNAs for NSCLC. The aim of this study was to identify differences in miRNA profiles of plasma exosome between NSCLC patients and healthy controls, and to further examine the prognostic role of exosomal miRNAs in NSCLC.

## RESULTS

### Characterization of isolated exosomes

Transmission electron microscope (TEM) indicated that isolated exosomes had a spherical shape with a diameter of about 30–100nm (Supplementary Figure 1). NanoSight analysis revealed that the size distribution of exosomes ranged from approximately 50nm to 150nm in diameter, with the mode value of 73nm (Supplementary Figure 2). The presence of exosomal markers, CD63 and CD9, was also confirmed by Western blot. The endoplasmic reticulum protein calnexin was absent in exosome and exosome-free plasma. Most of albumin was enriched in the exosome-free plasma, which was in accordance with previous reports [22–24] (Supplementary Figure 3).

### Exosomal miRNA profiling

We performed plasma exosomal miRNA profiling for 10 patients with lung adenocarcinoma and 10 matched healthy controls. The quantitative polymerase chain reaction (qPCR) panel was designed to quantify 84 cancer-related miRNAs. The panel data was analyzed with GenEx software as mentioned in MATERIALS AND METHODS. A total of 30 miRNAs were differentially expressed between lung adenocarcinoma patients and healthy controls (Supplementary Table 1), among which, 25 miRNAs were down-regulated and 5 miRNAs were up-regulated (P < 0.05, fold change > 1.5). Considering differential expression and previous reports regarding exosome and miRNA, nine miRNAs (miR-23b-3p, miR-10b-5p, miR-194-5p, let-7g-5p, miR-19a-3p, miR-141-3p, miR-21-5p, miR-1290, and miR-375) were chosen for further analyses of their prognostic values. Among them, exosomal miR-21-5p, miR-1290, and miR-375 levels showed prognostic significance in patients with glioma or prostate cancer [19, 25], and their expression in tumor tissue or blood correlated with overall survival in NSCLC patients [26–28]. Thus, these three exosomal miRNAs were also included to evaluate their prognostic significance in NSCLC.

### Endogenous reference gene determination

We analyzed the RNA profiling data to select candidate endogenous reference genes. SNORD49A had the smallest standard deviation of Cq among the profiled RNAs (Supplementary Figure 4A). Using the qPCR panel data, the web-based tool RefFinder identified let-7a-5p as the top ranking reference gene (Supplementary Figure 4B). Two exosomal miRNAs, miR-30a-5p and miR-30e-5p, were used as endogenous references in a published study [19]. The expression levels of these 4 candidate endogenous reference small RNAs (SNORD49A, let-7a-5p, miR-30a-5p, and miR-30e-5p) were further evaluated in 18 plasma samples. The quantification data of the 4 candidates was analyzed utilizing RefFinder. Let-7a-5p was identified as the top ranking reference candidate, followed sequentially by miR-30e-5p, SNORD49A and miR-30a-5p (Supplementary Figure 4C). Thus, let-7a-5p was selected as endogenous reference in the subsequent qPCR assay.

### Prognostic role of plasma exosomal miRNA expression levels in patients with NSCLC

To assess the potential prognostic role of exosomal miRNA expression levels, 9 miRNAs (miR-23b-3p, miR-10b-5p, miR-194-5p, let-7g-5p, miR-19a-3p, miR-141-3p, miR-21-5p, miR-1290, and miR-375) were further quantified in plasma from 209 NSCLC patients. MiR-141-3p was not detectable in 97% patients. Thus, miR-141-3p was removed from prognosis analysis. Besides, 13 patients with Cq of the endogenous reference greater than 37 were removed for analysis. Finally, the prognostic roles of only 8 miRNAs were analyzed in 196 NSCLC patients. Since miR-21-5p, miR-1290, and miR-375 were selected based on previous reports, their exosomal levels in 21 controls, including 10 healthy controls and 11 patients with non-tumor respiratory diseases, were also detected by qPCR. And miR-21-5p, miR-1290 and miR-375 were all up-regulated in NSCLC (Mann-Whitney U test, P = 0.000, 0.011, and 0.000, respectively; Fold change = 3.52, 3.61, and 3.60, respectively).

Demographic and clinical characteristics of the 196 patients are shown in Table 1. The median follow-up time was 14.40 months (range: 3.43 - 36.87 months), and 71 (36.22%) patients were dead at the end of study.

**Table 1:** Characteristics of NSCLC patients for survival analysis

Characteristics	Patients(N=196)
Follow-up time, median(range), month	14.40(3.43-36.87)
Age at diagnosis, mean ± SD, year	58.49 ± 9.95
Gender	
Male	137(69.90%)
Female	59(30.10%)
Smoking status	
Current smoker	103(52.55%)
Never smoker	66(33.67%)
Former smoker	27(13.78%)
Histological type	
Adenocarcinoma	115(58.67%)
Squamous cell carcinoma	73(37.24%)
Large cell carcinoma	3(1.53%)
Adenosquamous carcinoma	4(2.04%)
other NSCLC *	1(0.51%)
Pathological stage	
I-IIIa	100(51.02%)
IIIb-IV	87(44.39%)
Unknown	9(4.59%)
Chemotherapy	
Yes	124(63.27%)
No	72(36.73%)
Surgery treatment	
Yes	89(45.41%)
No	107(54.59%)
Radiotherapy	
Yes	11(5.61%)
No	185(94.39%)
History of COPD ^#^	
Yes	19(9.69%)
No	177(90.31%)

* Mucoepidermoid carcinoma.

# COPD, chronic obstructive pulmonary disease.

**Figure 1:**
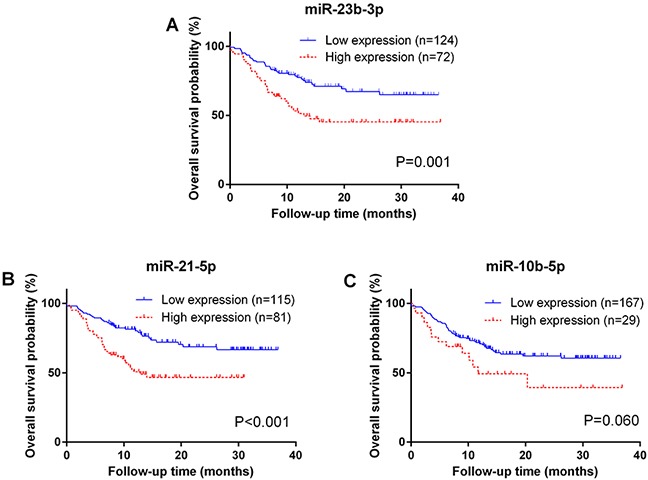
Survival analysis of plasma exosomal miRNA in non-small-cell lung cancer **A, B**. Kaplan-Meier curves show that relative expression levels of exosomal miR-23b-3p and miR-21-5p are significantly associated with overall survival (OS); **C**. miR-10b-5p shows a trend association with OS but not statistically significant. P-values were calculated using log-rank tests.

**Figure 2:**
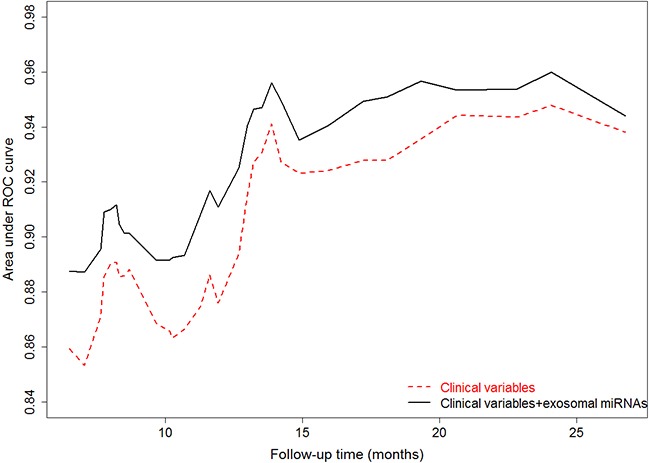
Area under the ROC curves (AUCs) based on multivariate Cox proportional hazard model using a time-dependent ROC analysis With addition of the 3-exosomal miRNA signature (miR-23b-3p, miR-10b-5p, and miR-21-5p), AUC is improved from 0.88 to 0.91 (P=0.015) at the time of 12-month. Clinical variables include smoking history, tumor stage, surgery and chemotherapy.

A backward selection process was used to screen potential confounders. Smoking status, tumor stage, chemotherapy and surgery were significant confounders, and were retained as covariates in all subsequent multivariable analyses (Supplementary Table 2). A multivariable COX proportional hazard analysis showed a poorer overall survival in patients with high expression levels of exosomal miR-23b-3p (P = 0.001; hazard ratio [HR] = 2.42, 95% CI: 1.45 - 4.04), miR-10b-5p (P = 0.013; HR = 2.22, 95% CI: 1.18 - 4.16) and miR-21-5p (P = 0.003; HR = 2.12, 95% CI: 1.28 - 3.49) (Table 2). Kaplan-Meier curves and log rank tests also demonstrated significant overall survival differences between the low and high expression levels of exosomal miR-23b-3p (P = 0.001) and miR-21-5p (P < 0.001) (Figure 1A, 1B). MiR-10b-5p showed a boardline level of significant association with OS (Figure 1C, P=0.060). In addition, time-dependent area under the receiver operating characteristic (ROC) curve (AUC) was generated to evaluate survival predictive accuracy (Figure 2). When compared to the clinical variables only model, adding the 3-miRNA signatures significantly improved AUC from 0.88 to 0.91 at 12-month follow-up time (P = 0.015) (Figure 2).

**Table 2:** Association of plasma exosomal miRNAs with overall survival in patients with NSCLC

Plasma exosomal miRNA	P-value	HR (95% CI)
miR-375	0.715	1.10 (0.65-1.88)
miR-23b-3p	0.001	2.42 (1.45-4.04)
miR-21-5p	0.003	2.12(1.28-3.49)
miR-19a-3p	0.096	1.60 (0.92-2.79
miR-194-5p	0.781	1.10 (0.56-2.17)
miR-1290	0.127	1.57 (0.88-2.81)
miR-10b-5p	0.013	2.22 (1.18-4.16)
let-7g-5p	0.360	1.29 (0.75-2.22)

Hazard ratios (HRs), 95% confidence intervals (CI), and p-values were calculated using a Cox proportional hazard model adjusting for smoking status, tumor stage, chemotherapy, and surgery treatment.

## DISCUSSION

This is the first study to investigate prognostic significance of exosomal miRNAs in NSCLC. Our findings demonstrated that plasma exosomal miR-23b-3p, miR-10b-5p and miR-21-5p could serve as independent prognostic biomarkers for NSCLC patients.

Exosomes play diverse roles in tumorigenesis and progression [29, 30]. Glioblastoma-derived exosomes could stimulate glioma cell proliferation and stimulate angiogenesis *in vitro* [31]. Kogure *et al*. found that hepatocellular carcinoma cell-derived exosomes enhanced cancer cell growth in recipient cells [32]. Exosomes derived from CD105-positive cancer stem cells could trigger angiogenesis, promote formation of a premetastatic niche and enhance lung metastasis formation [33]. It was reported that tumor-derived exosomes could enhance metastasis by bone marrow education [34], and that exosomes may participate in tumor metastasis via exosomal miRNAs [35, 36]. Exosomes also play important roles in stromal remodelling and immune regulation in cancer [37, 38].

MiR-21-5p is a known oncogenic miRNA [39, 40], and is up-regulated in tissues and serum of NSCLC patients [41–43]. Exosomal miR-21 levels in the cerebrospinal fluid of glioma patients were found to be significantly higher than that in the brain trauma patients, and correlated with tumor metastasis and recurrence [25]. Tanaka *et al*. confirmed that exosomal miR-21 was up-regulated in serum from patients with esophageal squamous cell cancer, and was associated with tumor progression and aggressiveness [18]. These lines of evidence, along with our results, indicate that exosomal miR-21-5p might be involved in tumor progression, and has the potential to be used as a prognostic biomarker in clinical applications, although its exact pathologic mechanism still needs to be elucidated.

It has been reported that miR-10b expression level was higher in NSCLC tissues compared with adjacent normal lung tissues, and that miR-10b-silenced cells showed up-regulation of apoptosis-inducing factors [44]. MiR-10b could also enhance pancreatic cancer cell invasion [45]. A recent study revealed that exosomal miR-10b was functional inside the recipient cells and could induce invasion ability in HMLE cells [46].

MiR-23b-3p is reported to be down-regulated [47] or up-regulated [48, 49] in NSCLC samples. MiR-23b-3p has been known to perform various roles in tumorigenesis and progression [49–54]. In fact, miR-23b-3p intriguingly plays opposite roles on the development of human cancers and regulation of reactive oxygen species (ROS) [55]. Ostenfeld *et al*. found that miR-23b acted as a tumor suppressor at different steps of the metastatic process in bladder cancer, and that cellular disposal of miR-23b by exosome release was linked to acquisition of metastatic properties [36]. Interestingly, one recent study suggested that exosomal transfer of miR-23b from the bone marrow may promote breast cancer cell dormancy in a metastatic niche [56], which was associated with breast cancer recurrence.

Liquid biopsy is a prominent research topic in precision medicine for cancer, due to its noninvasive property allowing repeated monitoring to gain a broader molecular understanding of tumors without the need for a tissue biopsy. Tumor-derived circulating exosomes have attracted increasing interest as a promising alternative to liquid biopsy. It was reported that the majority of miRNAs detectable in plasma is concentrated in exosomes [57]. There are potential advantages using exosomal miRNA as biomarker compared to total plasma miRNA. Firstly, miRNAs contained in exosomes are reflective of the status of parental cells [58, 59]. Secondly, the lipid bilayer membrane of exosomes protects miRNAs from degradation and guarantees their better stability for long-term storage. Thirdly, exosome reduces the complexity of biofluids thereby aiding in more specific and more sensitive detection of low abundance molecules [60].

Although our study revealed some interesting findings, there exist some limitations. One limitation was that the findings were not validated in an independent cohort. Secondly, mechanism of exosomal miRNA affecting prognosis was not explored. Since there exist some relevant publications, we systematically searched and reviewed the literatures on the role of target genes in tumorigenesis/metastasis and their expression in NSCLC. For target genes whose expression was not reported in lung cancer, we used a cancer microarray database Oncomine (https://www.oncomine.org) [61]. Abnormal expressions of target genes in NSCLC and their roles in tumorigenesis/metastasis were summarized in Supplementary Table 3. As for example, 14 target genes of miR-23b-3p were reported, including NISCH, FAS, PTEN, VHL, ATG12, ZEB1, SRC, AKT, NOTCH2, ETS1, SMAD3, HMGB2, TFAM and IRF1. Most target genes of miR-23b-3p, miR-10b-5p and miR-21-5p regulate epithelial-to-mesenchymal transition, growth, invasiveness, angiogenesis, and metastatic phenotypes of cancers (Supplementary Table 3).

In conclusion, plasma exosomal miR-23b-3p, miR-10b-5p and miR-21-5p were independent prognostic biomarkers of NSCLC; their expression levels provided a significantly improved survival prediction for NSCLC beyond conventional clinical predictors. However, further studies are needed to validate possible application of these exosomal miRNAs for survival prediction and to explore their potential mechanisms in lung cancer progression.

## MATERIALS AND METHODS

### Patients and samples

Patients with NSCLC newly diagnosed histologically were recruited from October 2012 to December 2014 at Xinqiao Hospital of the Third Military Medical University in Chongqing, China. The TNM stage of disease was defined according to the 7th edition of the International Association for the Study of Lung Cancer classification system. Patients were excluded if they had a history of heart, liver, and kidney disease, or diabetes mellitus. Controls were recruited from outpatients who had undergone a general medical examination; individuals with a history of any acute or chronic inflammatory disease and infectious disease were excluded, and those who had elevated serologic tumor markers and an abnormal chest examination (including ground glass opacity, fibrous stripes, and small nodules) were also excluded. History of chronic obstructive pulmonary disease (COPD) was determined based on explicit diagnosis recorded in the medical history. Never smokers were defined as individuals who had smoked less than 100 cigarettes during their lifetime. Former smokers were defined as individuals who had quit smoking at least one year at the survey time. Clinical and demographic information was obtained from each study subject by a combination of a structured subject interview and medical records. Blood (10ml) was collected for each patient. Plasma was separated immediately and stored at -80˚C before use. All patients were actively followed for the first time within 6 months of diagnosis, with subsequent annual follow-up by telephone interview. Annual verification of vital status of patients was accomplished with the use of electronic medical notes and next-of-kin reports. The study was approved by the Ethics Committee of Third Military Medical University, and all participants provided informed consent.

### Exosome isolation

Exosomes were isolated from plasma using ExoQuick Exosome Precipitation Solution kit (System Biosciences, Mountain View, CA, USA). Briefly, 3μL thrombin (System Biosciences, Mountain View, CA, USA) was added in 300μL plasma to eliminate fibrinogen. After 5min incubation while mixing, plasma was centrifuged at 17,000g for 10min. Then, 250μL serum-like supernatant was removed into a new tube and mixed with 63μL ExoQuick reagent. After incubation at 4˚C overnight, the mixture was centrifuged at 1,500g for 30min. The supernatant was removed, and the exosome pellet was again centrifuged at 1,500g for 5min to remove all traces of fluid. The pellet was then resuspended in 25μL PBS, which was filtrated with a 0.22μm syringe filter (Millipore, Billerica, MA, USA).

### Exosome characterization

Exosome was characterized with TEM, Western blot, and NanoSight analysis. For TEM, 2μL exosome suspension was diluted with 8μL PBS, and then adsorbed to copper-coated mesh-grids for 10 minutes. Redundant suspension was removed with filter paper, and the grids were allowed to dry. Exosomes were then negatively stained with 2% sodium phosphotungstate for 30 seconds. The samples were viewed with a JEM-1400Plus transmission electron microscope (JEOL, Tokyo, Japan).

The exosome markers, CD9 and CD63, were identified using Western blot. To extract protein, the exosome pellet was lysed in 200μL RIPA lysis buffer (Beyotime Biotechnology, Shanghai, China) containing 1 mM phenylmethylsulphonyl fluoride (Beyotime Biotechnology). The total exosomal protein content was measured using BCA protein assay kit (Beyotime Biotechnology). Protein (50μg) was separated by 10% SDS-PAGE, transferred to a PVDF membrane, blocked with 5% nonfat milk in PBS-Tween (0.1%). The PVDF membranes were then incubated with rabbit anti-CD9 (System Biosciences, Mountain View, CA, USA; dilution 1/1,000), rabbit anti-CD63 (Abcam, Shanghai, China; dilution 1/800), rabbit anti-albumin (Abcam, Shanghai, China; dilution 1/5000), or rabbit anti-calnexin (Abcam, Shanghai, China; dilution 1/5000) overnight at 4˚C, followed by incubation with goat anti-rabbit HRP secondary antibody (System Biosciences; dilution 1/20,000) for 1h at room temperature. The proteins were detected using enhanced chemiluminescence (Wanleibio, Shenyang, China).

Size distribution of exosome was analyzed using a NanoSight NS300 instrument (Malvern Instruments Ltd, Worcestershire, UK). For NanoSight analysis, the exosome pellet was resuspended in 500μL steriled PBS, which was filtrated with a 0.22μm syringe filter (Millipore). Samples were diluted (1:4000) until individual nanoparticles could be tracked.

### Exosomal RNA isolation

Exosomal RNA was isolated from exosome suspension using miRNeasy Micro Kit (Qiagen, Hilden, Germany). MiRNA content was quantified using Qubit^®^ microRNA Assay Kits (Invitrogen, Carlsbad, CA, USA), along with Qubit^®^ 2.0 Fluorometer (Invitrogen). The microRNA Assay Kits allows easy and accurate quantification of small RNA (~20 nucleotides or base pairs).

### Exosomal miRNA profiling

Exosomal miRNA was profiled using Cancer Focus microRNA PCR Panel (Exiqon, Vedbaek, Denmark). Each of the template RNA samples was adjusted to a concentration of 20-25ng in 14μL nuclease-free water. The 20μL reverse transcription system consisted of 4μL 5× Reaction buffer, 2μL Enzyme mix and 14μL template total RNA. The mixture was incubated for 60 min at 42˚C, followed by heat-inactivation of the reverse transcriptase for 5 min at 95˚C. The cDNA template was diluted 80× in nuclease-free water, and was then quantified using the Exiqon panel. SYBR^®^ Green master mix (5μL), PCR primer mix (1μL) and diluted cDNA template (4μL) was added into each well of the panel. Realtime PCR was performed for 10min at 95˚C, followed by 40 cycles of 10s at 95˚C and 1min at 60˚C (ramp-rate 1.6˚C/s), in which optical read was carried out. Melt curve analysis was done after the amplification cycles. Using the Exiqon PCR panel, exosomal miRNA in plasma was profiled in 10 patients with lung adenocarcinomas and 10 healthy controls matched by age, sex and history of smoking. The GenEx software (MultiD Analyses AB, Göteborg, Sweden) was utilized to analyze miRNA profiling data according to its Data Analysis Guide (http://www.multid.se/genex/genex.html).

### Endogenous reference gene determination

Currently, there is no consensus on endogenous control for exosomal miRNA qPCR assays [19, 62, 63]. According to previous reports and results from exosomal miRNA profiling, 4 exosomal small RNAs with stable expression were selected as candidate endogenous controls. Their expression was further determined using qPCR in 18 plasma samples (from 9 patients with NSCLC and 9 patients without tumor), and qPCR for each sample was performed in triplicate. For each sample, 1.0ng small RNA was used for reverse transcription with primer pool of the four candidate endogenous controls. The quantification data was analyzed with RefFinder, a user-friendly web-based tool [64]. RefFinder integrates NormFinder, geNorm, BestKeeper and comparative ΔCt methods, to compare and rank the tested candidate reference genes [64].

### Quantitative polymerase chain reaction

Exosomal RNA was reverse transcribed to cDNA with GoScript™ Reverse Transcription System (Promega, Madison, WI, USA) using 2μL 5μM stem-loop primer pool and 8μL exosomal RNA. The cDNA was diluted 1:4 and then quantified with SYBR^®^ Premix Ex Taq™ II (Takara, Dalian, China). In brief, a 25μL reaction was performed in a Bio-Rad CFX connect^TM^ Real-Time System using 12.5μL SYBR^®^ Premix Ex Taq II (Takara, Dalian, China), 1μL 10μM primer pair (Invitrogen, Shanghai, China), 2μL diluted cDNA, and 9.5μL DEPC-treated water. Realtime PCR was conducted for 30s at 95˚C, followed by 40 cycles of 5s at 95˚C and 30s at 60˚C. All samples were evaluated in duplicate, and all runs included non-template controls (NTC) for each miRNA analyzed. The Cq values for non-template controls (NTC) always exceeded 37. All reactions with Cq above 37 were considered negative. MiRNAs with more than 30% samples undetectable were omitted for analyses. PCR product purity was monitored by melting curve analysis and 2.0% agarose gel electrophoresis.

### Statistical analysis

The Exiqon qPCR panel data was pre-processed and analyzed using GenEx software as suggested by the instruction in the kit. Inter-plate calibration was performed to reduce run-to-run variation; then erroneous and missing Cq values were detected and deselected using outlier detection. Cq values more than 37 were deemed as background, and miRNAs with more than 40% samples undetected were omitted for analyses. NormFinder [65] implanted in GenEx software, which calculated accumulated standard deviation (Acc.SD), was employed to defined reference genes from candidate genes with all Cq < 34. Expression data was normalized with reference genes, and then converted to relative quantities and log_2_ transformed. A *t*-test was used to identify aberrantly expressed exosomal miRNAs in NSCLC.

For qPCR data analyses, each exosomal miRNA expression level was determined as low or high using cut-off identified by X-tile, which is a bio-informatics tool for outcome-based cut-point optimization [66]. As a graphical method, X-tile provides a single, global assessment of every possible way of dividing a population into low and high-level marker expression. First, candidate miRNA expression was normalized to endogenous reference using ΔCq method. To assess statistical significance and avoid the problems of multiple cut-point selection, we used the X-tile program to randomly divide the total cohort into two equal training and validation sets. “Optimal” cut-point was then selected based on corrected P values produced by standard Monte Carlo simulations.

A backward selection process was used to select potential confounders: age at diagnosis, gender, smoking status, stage, histologic types, chemotherapy, radiotherapy, surgery and history of COPD. The significant variables were retained as covariates in all subsequent multivariate analyses. A multivariate Cox proportional hazard model was performed to assess association between expression level of each exosomal miRNA and OS. The Kaplan-Meier method was used to generate survival curves, and survival rates were compared using the log-rank test. Time-dependent AUC was estimated and compared using method of Blanche *et al*. [67]. Statistical analyses were performed by using SPSS software package (Version 13.0, SPSS Inc., Chicago, IL, USA) or R package [67, 68]. Kaplan-Meier curves were generated using GraphPad Prism (Version 6.04 for Windows, SanDiego, CA, USA). All reported p values were two-sided with a significance level of 0.05.

## SUPPLEMENTARY FIGURES AND TABLES




