# Supplementary material for: Circulating exosomal microRNAs as prognostic biomarkers for non-small-cell lung cancer
Source: Oncotarget. 2016 Dec 30;8(8):13048–58. doi: 10.18632/oncotarget.14369 (PMC5355076; doi:10.18632/oncotarget.14369)
Supplement: Supplementary file 1 [file oncotarget-08-13048-s001.pdf]

Circulating exosomal microRNAs as prognostic biomarkers for non-small-cell lung cancer

SUPPLEMENTARY FIGURES AND TABLES

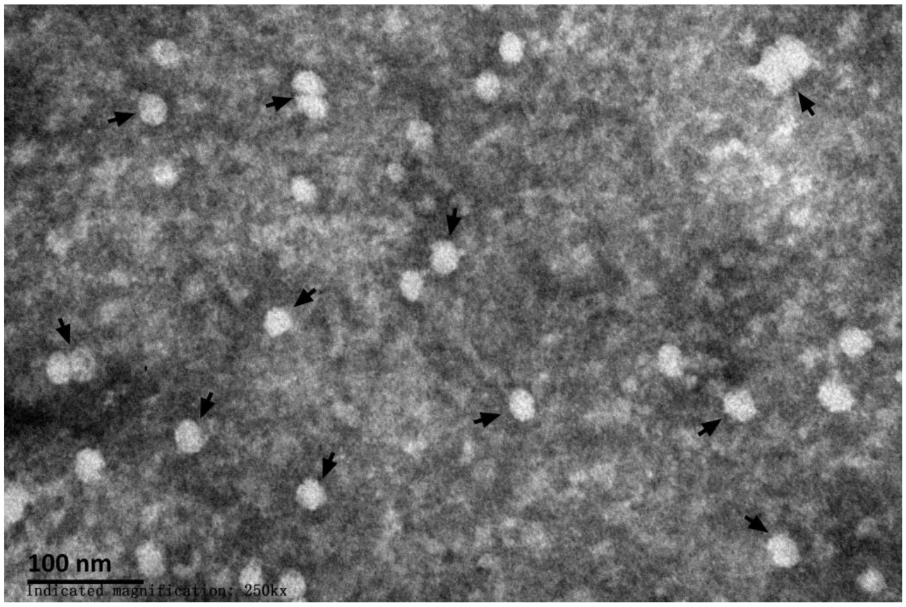

Supplementary Figure 1: Transmission electron microscope image for plasma exosomes in lung cancer patients. Arrows indicate exosomes.

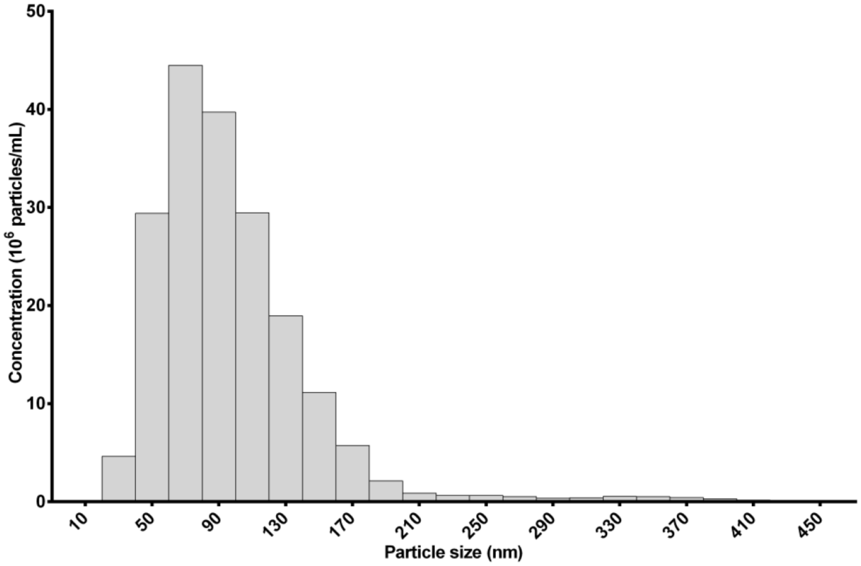

Supplementary Figure 2: NanoSight analysis for plasma exosomes in lung cancer patients. Horizontal axis, particle size (nm); vertical axis, particle concentration (10^6 particles/mL).

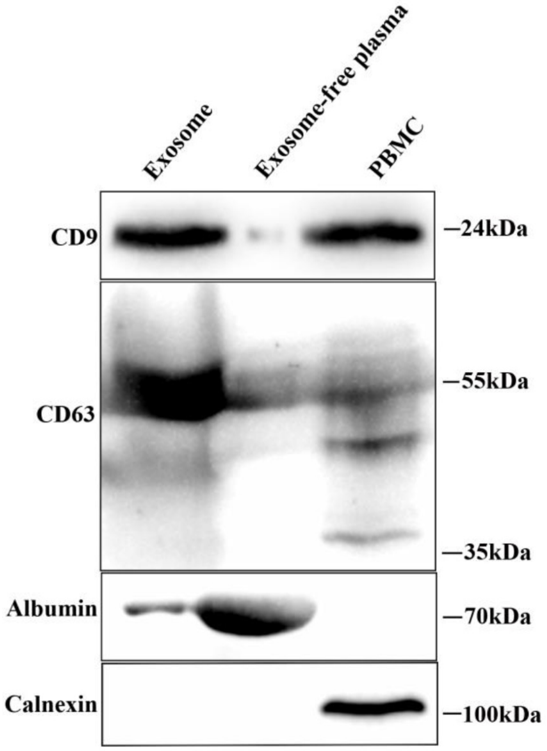

Supplementary Figure 3: Protein markers detection by Western blot. Exosome-free plasma, the part of plasma that exosome was depleted by ExoQuick reagent; PBMC, peripheral blood mononuclear cell.

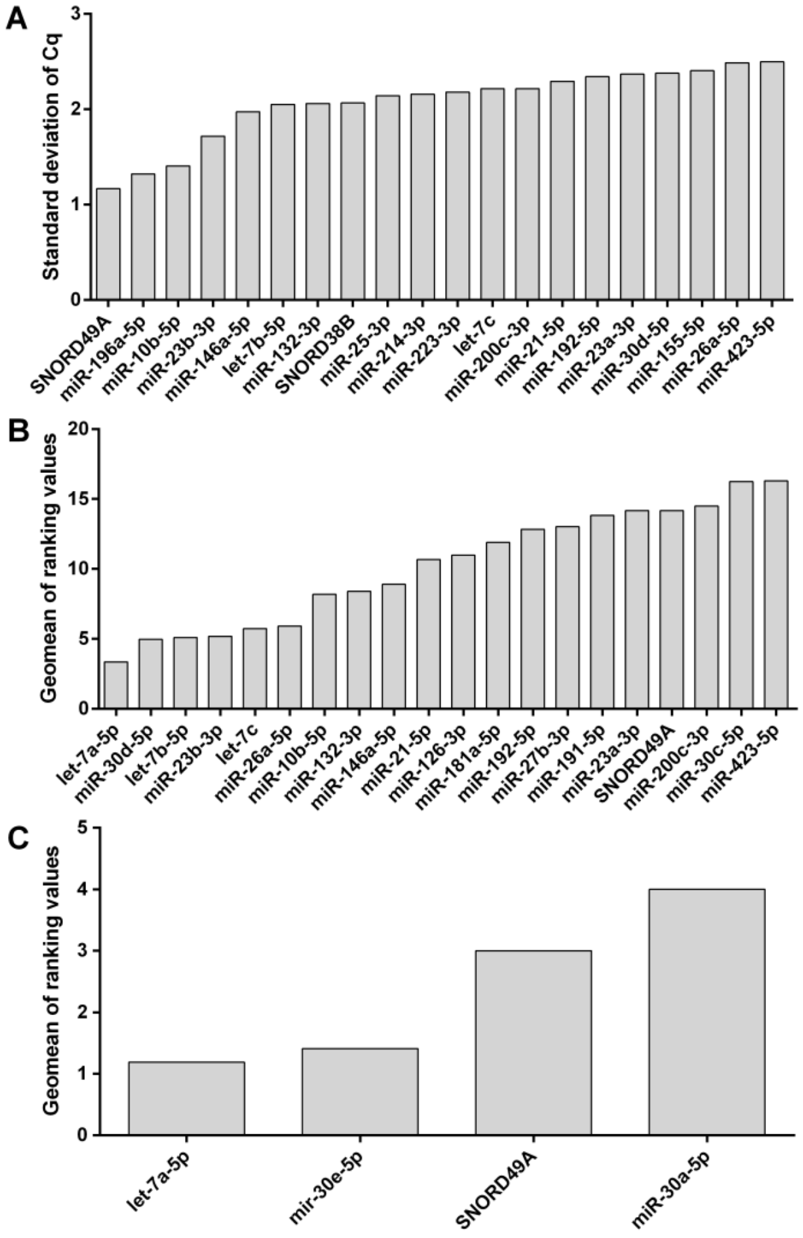

Supplementary Figure 4: Evaluations of miRNA candidates for endogenous normalization. A. top 20 genes with the lowest standard deviation for the qPCR panel data. **B.** top 20 genes with the lowest geomean of ranking values identified by RefFinder (http://fulxie.0fees.us/?type=reference&ckattempt=1) using qPCR panel data. Based on the rankings from each program (geNorm, NormFinder, BestKeeper, and the comparative delta Ct method), RefFinder assigns an appropriate weight to an individual gene and calculates the geometric mean of their weights for the overall final ranking. A lower geomean of ranking value means better stability. **C.** geomean of ranking values of 4 candidate endogenous reference genes identified by RefFinder.

Supplementary Table 1: The 30 abnormally expressed miRNAs in lung adenocarcinoma patients identified by the qPCR panel

miRNAs	Fold change*	P-value#
let-7i-5p	-7.26	<0.0001
miR-22-3p	-6.54	<0.0001
miR-19b-3p	-22.70	0.001
miR-186-5p	-4.28	0.001
miR-29a-3p	-7.49	0.001
miR-19a-3p	-2.45	0.001
miR-130a-3p	-2.79	0.001
miR-29b-3p	-3.82	0.002
miR-93-5p	-8.00	0.002
miR-20a-5p	-5.42	0.004
miR-30b-5p	-6.99	0.004
miR-23b-3p	1.99	0.004
let-7g-5p	-4.41	0.004
miR-101-3p	-2.74	0.005
miR-143-3p	-7.04	0.005
miR-9-5p	4.32	0.006
miR-107	-3.16	0.007
miR-141-3p	-3.78	0.008
miR-10b-5p	2.25	0.008
miR-106b-5p	-3.90	0.012
miR-103a-3p	-5.45	0.017
miR-106a-5p	-4.67	0.018
miR-132-3p	2.41	0.020
miR-34a-5p	-2.92	0.022
miR-194-5p	-3.36	0.022
miR-210	-3.26	0.022
let-7d-5p	-2.18	0.032
miR-27b-3p	-2.29	0.037
miR-30d-5p	-1.75	0.040
miR-196a-5p	2.80	0.046

*Fold change of lung adenocarcinoma patients relative to healthy controls.
#P-value was calculated with unpaired *t*-test using GenEx software

Supplementary Table 2: Significant clinical variables associated with overall survival in patients with non-small cell lung cancer

Variables*	HR	95% CI of HR	P-value
Smoking status			0.004
Never smoker	Reference		
Current smoker	1.64	0.92-2.93	0.093
Former smoker	3.49	1.67-7.29	0.001
Pathological stage			0.011
I-IIIa	Reference		
IIIb-IV	3.14	1.22-8.06	0.018
Unknown	1.12	0.31-4.13	0.862
Chemotherapy	2.71	1.60-4.58	<0.0001
Surgery treatment	5.24	1.62-16.89	0.006

*Variables identified using a stepwise Cox proportional hazard model (Backward: LR, Entry: 0.05; Removal: 0.10).

Supplementary Table 3: Target gene expression of miR-23b-3p, miR-10b-5p and miR-21-5p in non-small cell lung cancer and their functions.

See Supplementary File 1
