# Supplementary material for: Circulating exosomal microRNAs as prognostic biomarkers for non-small-cell lung cancer
Source: Oncotarget. 2016 Dec 30;8(8):13048–58. doi: 10.18632/oncotarget.14369 (PMC5355076; doi:10.18632/oncotarget.14369)
Supplement: Supplementary file 2 [file oncotarget-08-13048-s002.doc]

**Supplementary Table S3** Target gene expression of miR-23b-3p, miR-10b-5p and miR-21-5p in non-small cell lung cancer and their functions

| **miRNAs** | **Main target genes** | **Expression in NSCLC** | **Functions in tumorigenesis/metastasis** | **References** |
| --- | --- | --- | --- | --- |
| **miR-23b-3p** | NISCH | Downregulation^#^ | Inhibit growth, proliferation, migration, invasion, and metastasis |  |
|  | FAS | Downregulation | Induce apoptosis; inhibit cisplatin tolerance |  |
|  | PTEN | Downregulation | Negatively regulate PI3K/AKT pathway; inhibit proliferation, invasion, metastasis, drug resistance, and radioresistance |  |
|  | VHL | Downregulation | Oxygen-dependent polyubiquitylation of hypoxia-inducible factor; upregulate p53 |  |
|  | ATG12 | Upregulation^#^ | An autophagy-related protein; a determinant of primary resistance to HER2-targeted therapies; a positive mediator of mitochondrial apoptosis |  |
|  | ZEB1 | Upregulation | Induce epithelial-to-mesenchymal transition; promote growth, motility, invasiveness, and metastatic phenotypes |  |
|  | SRC | Upregulation | Provide oncogenic signals for cell survival, mitogenesis, epithelial-to-mesenchymal transition, invasion, angiogenesis, and metastasis |  |
|  | AKT | Upregulation | Promote cell survival, proliferation, metastasis, and angiogenesis |  |
|  | NOTCH2 | Downregulation  /upregulation | Promote growth, invasion, epithelial-to-mesenchymal transition, and metastasis; regulate apoptosis |  |
|  | ETS1 | Upregulation | Promote migration, epithelial-to-mesenchymal transition, invasive, angiogenesis, drug resistance, and apoptosis; inhibit tumorigenicity |  |
|  | SMAD3 | Upregulation | Inhibiting cell proliferation, promote apoptosis; promote epithelial-to-mesenchymal transition, invasion, and metastasis |  |
|  | HMGB2 | Upregulation^#^ | Protects cell from DNA damage; inhibit chemo- and radio-sensitivity |  |
|  | TFAM | Upregulation | Promote growth and migration; inhibit apoptosis and chemosensitivity |  |
|  | IRF1 | Downregulation^#^ | Promote apoptosis |  |
| **miR-10b-5p** | HOXD10 | Upregulation^#^ | Promote proliferation; regulate migration and invasion |  |
|  | HOXB3 | NA | Promote apoptosis and cisplatin chemosensitivity; regulate migration, invasion, and proliferation |  |
|  | TIAM1 | Upregulation | Promote proliferation, tumorigenicity, migration, invasion, and metastasis while induce cell adhesion |  |
|  | NR5A2 | Downregulation^#^ | Promote proliferation, sphere formation, migration, invasion, metastasis, and angiogenesis |  |
|  | BCL2L11 | Upregulation^#^ | Initiate apoptosis |  |
|  | TFAP2C | Upregulation | Promote proliferation, tumorigenesis, and aggressiveness |  |
|  | CDKN1A | Upregulation | Negative regulator of the cell cycle; inhibit apoptosis |  |
|  | CDKN2A | Downregulation | Regulate cell cycle and senescence |  |
|  | KLF4 | Downregulation | Inhibit invasion, proliferation, and metastasis; promote apoptosis |  |
|  | PTEN | Downregulation | Negatively regulate PI3K/AKT pathway; inhibit proliferation, invasion, metastasis, drug resistance, and radioresistance |  |
|  | TBX5 | Downregulation^#^ | Inhibit proliferation, migration, invasion, and metastasis; induce apoptosis |  |
|  | ZEB1 | Upregulation | Induce epithelial-to-mesenchymal transition, increase motility and invasiveness, promote growth and metastatic phenotypes |  |
|  | PIK3CA | Upregulation | Promote proliferation, epithelial-to-mesenchymal transition, invasiveness, metastasis, and tumorigenicity ; inhibit apoptosis |  |
|  | SDC1 | Downregulation | Inhibit apoptosis; promote angiogenesis; regulate proliferation, migration, invasion, and metastasis |  |
| **miR-21-5p** | PTEN | Downregulation | Negatively regulate PI3K/AKT pathway; inhibit proliferation, invasion, metastasis, drug resistance, and radioresistance |  |
|  | PDCD4 | Downregulation | Inhibit growth, proliferation, invasion, and cell cycle transition; induce apoptosis |  |
|  | TIMP3 | Downregulation | Inhibit growth, angiogenesis, migration, invasion, and cisplatin resistance; induce apoptosis |  |
|  | TPM1 | Downregulation^#^ | Inhibit cell viability, migration, invasion, and radiation resistance; promote apoptosis |  |
|  | RECK | Downregulation | Inhibit vascular sprouting and angiogenesis |  |
|  | MSH2 | Downregulation | DNA mismatch repair; inhibit cell growth |  |
|  | CDC25A | Upregulation | Cell cycle regulator |  |
|  | FASL | Defect expression | Regulator of apoptosis |  |
|  | BTG2 | Downregulation^#^ | Inhibit proliferation and apoptosis; regulate migration and invasiveness |  |

This table was summarized by literature reviewing and Oncomine ( http://www.oncomine.org ).

^#^Data was from Oncomine, with fold change>1.5 and P<0.05.

NSCLC, non-small cell lung cancer; NA, abnormal expression of target gene was not reported in non-small cell lung cancer.

**References**

1. Jin LJ, Wessely O, Marcusson EG, Ivan C, Calin GA and Alahari SK. Prooncogenic Factors miR-23b and miR-27b Are Regulated by Her2/Neu, EGF, and TNF-alpha in Breast Cancer. Cancer Research. 2013; 73:2884-2896.

2. Baranwal S, Wang Y, Rathinam R, Lee J, Jin L, McGoey R, Pylayeva Y, Giancotti F, Blobe GC and Alahari SK. Molecular characterization of the tumor-suppressive function of nischarin in breast cancer. J Natl Cancer Inst. 2011; 103:1513-1528.

3. Alahari SK, Lee JW and Juliano RL. Nischarin, a novel protein that interacts with the integrin alpha5 subunit and inhibits cell migration. J Cell Biol. 2000; 151:1141-1154.

4. Li J, He X, Dong R, Wang Y, Yu J and Qiu H. Frequent Loss of NISCH Promotes Tumor Proliferation and Invasion in Ovarian Cancer via Inhibiting the FAK Signal Pathway. Mol Cancer Ther. 2015; 14:1202-1212.

5. Li B, Sun M, Gao F, Liu W, Yang Y, Liu H, Cheng Y, Liu C and Cai J. Up-regulated expression of miR-23a/b targeted the pro-apoptotic Fas in radiation-induced thymic lymphoma. Cell Physiol Biochem. 2013; 32:1729-1740.

6. Yi Y, Gan X, Lin C and Hu X. [Expression and significance of Fas/APO-1 and Bcl-2 protein in human lung cancer.]. Zhongguo Fei Ai Za Zhi. 1999; 2:17-19.

7. Wang DF, Zeng CG, Lin YB, Hou JH and Zhu ZH. [Expression and clinical significance of apoptosis-related oncogenes in stage I-II non-small cell lung cancer]. Ai Zheng. 2006; 25:359-362.

8. O'Brien DI, Nally K, Kelly RG, O'Connor TM, Shanahan F and O'Connell J. Targeting the Fas/Fas ligand pathway in cancer. Expert Opin Ther Targets. 2005; 9:1031-1044.

9. Wu W, Wang HD, Guo W, Yang K, Zhao YP, Jiang YG and He P. Up-regulation of Fas reverses cisplatin resistance of human small cell lung cancer cells. J Exp Clin Cancer Res. 2010; 29:49.

10. Zaman MS, Thamminana S, Shahryari V, Chiyomaru T, Deng G, Saini S, Majid S, Fukuhara S, Chang I, Arora S, Hirata H, Ueno K, Singh K, et al. Inhibition of PTEN gene expression by oncogenic miR-23b-3p in renal cancer. PLoS One. 2012; 7:e50203.

11. Marsit CJ, Zheng SC, Aldape K, Hinds PW, Nelson HH, Wiencke JK and Kelsey KT. PTEN expression in non-small-cell lung cancer: evaluating its relation to tumor characteristics, allelic loss, and epigenetic alteration. Hum Pathol. 2005; 36:768-776.

12. Wang J, Chen H, Liao Y, Chen N, Liu T, Zhang H and Zhang H. Expression and clinical evidence of miR-494 and PTEN in non-small cell lung cancer. Tumor Biol. 2015; 36:6965-6972.

13. Tang JM, He QY, Guo RX and Chang XJ. Phosphorylated Akt overexpression and loss of PTEN expression in non-small cell lung cancer confers poor prognosis. Lung Cancer. 2006; 51:181-191.

14. Hollander MC, Blumenthal GM and Dennis PA. PTEN loss in the continuum of common cancers, rare syndromes and mouse models. Nat Rev Cancer. 2011; 11:289-301.

15. Maehama T and Dixon JE. The tumor suppressor, PTEN/MMAC1, dephosphorylates the lipid second messenger, phosphatidylinositol 3,4,5-trisphosphate. J Biol Chem. 1998; 273:13375-13378.

16. Zhang JG, Wang JJ, Zhao F, Liu Q, Jiang K and Yang GH. MicroRNA-21 (miR-21) represses tumor suppressor PTEN and promotes growth and invasion in non-small cell lung cancer (NSCLC). Clin Chim Acta. 2010; 411:846-852.

17. Davidson L, Maccario H, Perera NM, Yang X, Spinelli L, Tibarewal P, Glancy B, Gray A, Weijer CJ, Downes CP and Leslie NR. Suppression of cellular proliferation and invasion by the concerted lipid and protein phosphatase activities of PTEN. Oncogene. 2010; 29:687-697.

18. Liu ZL, Wang H, Liu J and Wang ZX. MicroRNA-21 (miR-21) expression promotes growth, metastasis, and chemo- or radioresistance in non-small cell lung cancer cells by targeting PTEN. Mol Cell Biochem. 2013; 372:35-45.

19. Sos ML, Koker M, Weir BA, Heynck S, Rabinovsky R, Zander T, Seeger JM, Weiss J, Fischer F, Frommolt P, Michel K, Peifer M, Mermel C, et al. PTEN loss contributes to erlotinib resistance in EGFR-mutant lung cancer by activation of Akt and EGFR. Cancer Res. 2009; 69:3256-3261.

20. Chen L, Han L, Zhang K, Shi Z, Zhang J, Zhang A, Wang Y, Song Y, Li Y, Jiang T, Pu P, Jiang C and Kang C. VHL regulates the effects of miR-23b on glioma survival and invasion via suppression of HIF-1alpha/VEGF and beta-catenin/Tcf-4 signaling. Neuro Oncol. 2012; 14:1026-1036.

21. Peng Z, Shan C and Wang H. [Expression of VHL and HIF-1alpha and its clinical significance in the lung cancer tissue]. Zhong Nan Da Xue Xue Bao Yi Xue Ban. 2009; 34:331-334.

22. Kim WY and Kaelin WG. Role of VHL gene mutation in human cancer. J Clin Oncol. 2004; 22:4991-5004.

23. Roe JS, Kim H, Lee SM, Kim ST, Cho EJ and Youn HD. p53 stabilization and transactivation by a von Hippel-Lindau protein. Mol Cell. 2006; 22:395-405.

24. Wang P, Zhang J, Zhang L, Zhu Z, Fan J, Chen L, Zhuang L, Luo J, Chen H, Liu L, Chen Z and Meng Z. MicroRNA 23b regulates autophagy associated with radioresistance of pancreatic cancer cells. Gastroenterology. 2013; 145:1133-1143 e1112.

25. An Y, Zhang Z, Shang Y, Jiang X, Dong J, Yu P, Nie Y and Zhao Q. miR-23b-3p regulates the chemoresistance of gastric cancer cells by targeting ATG12 and HMGB2. Cell Death Dis. 2015; 6:e1766.

26. Geng JF and Klionsky DJ. The Atg8 and Atg12 ubiquitin-like conjugation systems in macroautophagy. EMBO Rep. 2008; 9:859-864.

27. Cufi S, Vazquez-Martin A, Oliveras-Ferraros C, Corominas-Faja B, Urruticoechea A, Martin-Castillo B and Menendez JA. Autophagy-related gene 12 (ATG12) is a novel determinant of primary resistance to HER2-targeted therapies: utility of transcriptome analysis of the autophagy interactome to guide breast cancer treatment. Oncotarget. 2012; 3:1600-1614.

28. Rubinstein AD, Eisenstein M, Ber Y, Bialik S and Kimchi A. The autophagy protein Atg12 associates with antiapoptotic Bcl-2 family members to promote mitochondrial apoptosis. Mol Cell. 2011; 44:698-709.

29. Majid S, Dar AA, Saini S, Deng G, Chang I, Greene K, Tanaka Y, Dahiya R and Yamamura S. MicroRNA-23b functions as a tumor suppressor by regulating Zeb1 in bladder cancer. PLoS One. 2013; 8:e67686.

30. Li YJ, Ping C, Tang J and Zhang W. MicroRNA-455 suppresses non-small cell lung cancer through targeting ZEB1. Cell Biol Int. 2016; 40:621-628.

31. Larsen JE, Nathan V, Osborne JK, Farrow RK, Deb D, Sullivan JP, Dospoy PD, Augustyn A, Hight SK, Sato M, Girard L, Behrens C, Wistuba, II, et al. ZEB1 drives epithelial-to-mesenchymal transition in lung cancer. J Clin Invest. 2016; 126:3219-3235.

32. Majid S, Dar AA, Saini S, Arora S, Shahryari V, Zaman MS, Chang I, Yamamura S, Tanaka Y, Deng G and Dahiya R. miR-23b represses proto-oncogene Src kinase and functions as methylation-silenced tumor suppressor with diagnostic and prognostic significance in prostate cancer. Cancer Res. 2012; 72:6435-6446.

33. Ceppi P, Rapa I, Lo Iacono M, Righi L, Giorcelli J, Pautasso M, Bille A, Ardissone F, Papotti M and Scagliotti GV. Expression and pharmacological inhibition of thymidylate synthase and Src kinase in nonsmall cell lung cancer. Int J Cancer. 2012; 130:1777-1786.

34. Guarino M. Src signaling in cancer invasion. J Cell Physiol. 2010; 223:14-26.

35. Yuan Y, Du W, Wang Y, Xu C, Wang J, Zhang Y, Wang H, Ju J, Zhao L, Wang Z, Lu Y, Cai B and Pan Z. Suppression of AKT expression by miR-153 produced anti-tumor activity in lung cancer. Int J Cancer. 2015; 136:1333-1340.

36. Osaki M, Oshimura M and Ito H. PI3K-Akt pathway: its functions and alterations in human cancer. Apoptosis. 2004; 9:667-676.

37. Jiang BH and Liu LZ. AKT signaling in regulating angiogenesis. Curr Cancer Drug Targets. 2008; 8:19-26.

38. Liu Y, Chen LH, Yuan YW, Li QS, Sun AM and Guan J. Activation of AKT is associated with metastasis of nasopharyngeal carcinoma. Tumour Biol. 2012; 33:241-245.

39. Sheng S, Qiao M and Pardee AB. Metastasis and AKT activation. J Cell Physiol. 2009; 218:451-454.

40. Yin S, Wang P, Yang L, Liu Y, Wang Y, Liu M, Qi Z, Meng J, Shi TY, Yang G and Zang R. Wip1 suppresses ovarian cancer metastasis through the ATM/AKT/Snail mediated signaling. Oncotarget. 2016; 7:29359-29370.

41. Huang TT, Ping YH, Wang AM, Ke CC, Fang WL, Huang KH, Lee HC, Chi CW and Yeh TS. The reciprocal regulation loop of Notch2 pathway and miR-23b in controlling gastric carcinogenesis. Oncotarget. 2015; 6:18012-18026.

42. Baumgart A, Mazur PK, Anton M, Rudelius M, Schwamborn K, Feuchtinger A, Behnke K, Walch A, Braren R, Peschel C, Duyster J, Siveke JT and Dechow T. Opposing role of Notch1 and Notch2 in a Kras(G12D)-driven murine non-small cell lung cancer model. Oncogene. 2015; 34:578-588.

43. Huang YC, Niu HT, Zhao GQ and Lei YJ. Expression of Notch1 and Notch2 in Xuanwei Female Patients with Lung Cancer and Its Clinical Significance. J Thorac Oncol. 2015; 10:S727-S727.

44. Hayashi T, Gust KM, Wyatt AW, Goriki A, Jager W, Awrey S, Li N, Oo HZ, Altamirano-Dimas M, Buttyan R, Fazli L, Matsubara A and Black PC. Not all NOTCH Is Created Equal: The Oncogenic Role of NOTCH2 in Bladder Cancer and Its Implications for Targeted Therapy. Clin Cancer Res. 2016; 22:2981-2992.

45. Galic V, Shawber C, Shah M, Wright J, Herzog T, Kitajewski J and Tong G. NOTCH2 as a tumor suppressor in epithelial ovarian cancer. Gynecologic Oncology. 2012; 125, Supplement 1:S134.

46. O'Neill CF, Urs S, Cinelli C, Lincoln A, Nadeau RJ, Leon R, Toher J, Mouta-Bellum C, Friesel RE and Liaw L. Notch2 signaling induces apoptosis and inhibits human MDA-MB-231 xenograft growth. Am J Pathol. 2007; 171:1023-1036.

47. Rosati E, Sabatini R, Rampino G, Tabilio A, Di Ianni M, Fettucciari K, Bartoli A, Coaccioli S, Screpanti I and Marconi P. Constitutively activated Notch signaling is involved in survival and apoptosis resistance of B-CLL cells. Blood. 2009; 113:856-865.

48. Song WJ, Wang XY and Sun CY. Biological significance and the related molecular mechanism of Ets1 mRNA expression in lung cancer by tissue microarray (TMA). Chinese Journal of Cancer Research. 2007; 19:176-183.

49. Furlan A, Vercamer C, Desbiens X and Pourtier A. Ets-1 triggers and orchestrates the malignant phenotype of mammary cancer cells within their matrix environment. J Cell Physiol. 2008; 215:782-793.

50. Dittmer J. The role of the transcription factor Ets1 in carcinoma. Semin Cancer Biol. 2015; 35:20-38.

51. Pei H, Li C, Adereth Y, Hsu T, Watson DK and Li R. Caspase-1 is a direct target gene of ETS1 and plays a role in ETS1-induced apoptosis. Cancer Res. 2005; 65:7205-7213.

52. Suzuki H, Romano-Spica V, Papas TS and Bhat NK. ETS1 suppresses tumorigenicity of human colon cancer cells. Proc Natl Acad Sci U S A. 1995; 92:4442-4446.

53. Leone V, D'Angelo D, Pallante P, Croce CM and Fusco A. Thyrotropin regulates thyroid cell proliferation by up-regulating miR-23b and miR-29b that target SMAD3. J Clin Endocrinol Metab. 2012; 97:3292-3301.

54. Hu H, Xu Z, Li C, Xu C, Lei Z, Zhang HT and Zhao J. MiR-145 and miR-203 represses TGF-beta-induced epithelial-mesenchymal transition and invasion by inhibiting SMAD3 in non-small cell lung cancer cells. Lung Cancer. 2016; 97:87-94.

55. Millet C and Zhang YE. Roles of Smad3 in TGF-beta signaling during carcinogenesis. Crit Rev Eukaryot Gene Expr. 2007; 17:281-293.

56. Yanagisawa K, Osada H, Masuda A, Kondo M, Saito T, Yatabe Y, Takagi K and Takahashi T. Induction of apoptosis by Smad3 and down-regulation of Smad3 expression in response to TGF-beta in human normal lung epithelial cells. Oncogene. 1998; 17:1743-1747.

57. Yamazaki K, Masugi Y, Effendi K, Tsujikawa H, Hiraoka N, Kitago M, Shinoda M, Itano O, Tanabe M, Kitagawa Y and Sakamoto M. Upregulated SMAD3 promotes epithelial-mesenchymal transition and predicts poor prognosis in pancreatic ductal adenocarcinoma. Lab Invest. 2014; 94:683-691.

58. Xue J, Lin X, Chiu WT, Chen YH, Yu G, Liu M, Feng XH, Sawaya R, Medema RH, Hung MC and Huang S. Sustained activation of SMAD3/SMAD4 by FOXM1 promotes TGF-beta-dependent cancer metastasis. J Clin Invest. 2014; 124:564-579.

59. Shin YJ, Kim MS, Lee J, Kang M and Jeong JH. High-mobility group box 2 (HMGB2) modulates radioresponse and is downregulated by p53 in colorectal cancer cell. Cancer Biol Ther. 2013; 14:213-221.

60. Syed N, Chavan S, Sahasrabuddhe NA, Renuse S, Sathe G, Nanjappa V, Radhakrishnan A, Raja R, Pinto SM, Srinivasan A, Prasad TSK, Srikumar K, Gowda H, et al. Silencing of high-mobility group box 2 (HMGB2) modulates cisplatin and 5-fluorouracil sensitivity in head and neck squamous cell carcinoma. Proteomics. 2015; 15:383-393.

61. Jiang J, Yang J, Wang Z, Wu G and Liu F. TFAM is directly regulated by miR-23b in glioma. Oncol Rep. 2013; 30:2105-2110.

62. Xie D, Wu X, Lan L, Shangguan F, Lin X, Chen F, Xu S, Zhang Y, Chen Z, Huang K, Wang R, Wang L, Song X, et al. Downregulation of TFAM inhibits the tumorigenesis of non-small cell lung cancer by activating ROS-mediated JNK/p38MAPK signaling and reducing cellular bioenergetics. Oncotarget. 2016; 7:11609-11624.

63. Li Z, Chen B, Feng M, Ouyang H, Zheng M, Ye Q, Nie Q and Zhang X. MicroRNA-23b Promotes Avian Leukosis Virus Subgroup J (ALV-J) Replication by Targeting IRF1. Sci Rep. 2015; 5:10294.

64. Armstrong MJ, Stang MT, Liu Y, Yan J, Pizzoferrato E and Yim JH. IRF-1 inhibits NF-kappaB activity, suppresses TRAF2 and cIAP1 and induces breast cancer cell specific growth inhibition. Cancer Biol Ther. 2015; 16:1029-1041.

65. Kim PKM, Armstrong M, Liu Y, Yan P, Bucher B, Zuckerbraun BS, Gambotto A, Billiar TR and Yim JH. IRF-1 expression induces apoptosis and inhibits tumor growth in mouse mammary cancer cells in vitro and in vivo. Oncogene. 2004; 23:1125-1135.

66. Wang YF, Li Z, Zhao XH, Zuo XM and Peng ZH. miR-10b promotes invasion by targeting HOXD10 in colorectal cancer. Oncol Lett. 2016; 12:488-494.

67. Ma L, Teruya-Feldstein J and Weinberg RA. Tumour invasion and metastasis initiated by microRNA-10b in breast cancer. Nature. 2007; 449:682-688.

68. Liu Z, Zhu JM, Cao H, Ren H and Fang XD. miR-10b promotes cell invasion through RhoC-AKT signaling pathway by targeting HOXD10 in gastric cancer. Int J Oncol. 2012; 40:1553-1560.

69. Nakayama I, Shibazaki M, Yashima-Abo A, Miura F, Sugiyama T, Masuda T and Maesawa C. Loss of HOXD10 expression induced by upregulation of miR-10b accelerates the migration and invasion activities of ovarian cancer cells. Int J Oncol. 2013; 43:63-71.

70. Liu Z, Zhu J, Cao H, Ren H and Fang X. miR-10b promotes cell invasion through RhoC-AKT signaling pathway by targeting HOXD10 in gastric cancer. Int J Oncol. 2012; 40:1553-1560.

71. Sharpe DJ, Orr KS, Moran M, White SJ, McQuaid S, Lappin TR, Thompson A and James JA. POU2F1 activity regulates HOXD10 and HOXD11 promoting a proliferative and invasive phenotype in head and neck cancer. Oncotarget. 2014; 5:8803-8815.

72. Chen H, Fan YJ, Xu WS, Chen JY, Xu CH, Wei XN, Fang D and Feng Y. miR-10b Inhibits Apoptosis and Promotes Proliferation and Invasion of Endometrial Cancer Cells via Targeting HOXB3. Cancer Biother Radiopharm. 2016; 31:225-231.

73. Yang D, Yan R, Zhang X, Zhu Z, Wang C and Liang C. Deregulation of MicroRNA-375 inhibits cancer proliferation migration and chemosensitivity in pancreatic cancer through the association of HOXB3. Am J Transl Res. 2016; 8:1551-1559.

74. Chen J, Zhu S, Jiang N, Shang Z, Quan C and Niu Y. HoxB3 promotes prostate cancer cell progression by transactivating CDCA3. Cancer Lett. 2013; 330:217-224.

75. Palakurthy RK, Wajapeyee N, Santra MK, Gazin C, Lin L, Gobeil S and Green MR. Epigenetic silencing of the RASSF1A tumor suppressor gene through HOXB3-mediated induction of DNMT3B expression. Mol Cell. 2009; 36:219-230.

76. Moriarty CH, Pursell B and Mercurio AM. miR-10b Targets Tiam1 IMPLICATIONS FOR Rac ACTIVATION AND CARCINOMA MIGRATION. J Biol Chem. 2010; 285:20541-20546.

77. Wang HM and Wang J. Expression of Tiam1 in lung cancer and its clinical significance. Asian Pac J Cancer Prev. 2012; 13:613-615.

78. Boissier P and Huynh-Do U. The guanine nucleotide exchange factor Tiam1: a Janus-faced molecule in cellular signaling. Cell Signal. 2014; 26:483-491.

79. Minard ME, Kim LS, Price JE and Gallick GE. The role of the guanine nucleotide exchange factor Tiam1 in cellular migration, invasion, adhesion and tumor progression. Breast Cancer Res Treat. 2004; 84:21-32.

80. Huang J, Ye XH, Guan J, Chen B, Li QS, Zheng XK, Liu LY, Wang S, Ding YQ, Ding Y and Chen LH. Tiam1 is associated with hepatocellular carcinoma metastasis. Int J Cancer. 2013; 132:90-100.

81. Liu N, Tang LL, Sun Y, Cui RX, Wang HY, Huang BJ, He QM, Jiang W and Ma J. MiR-29c suppresses invasion and metastasis by targeting TIAM1 in nasopharyngeal carcinoma. Cancer Lett. 2013; 329:181-188.

82. Li Z, Yu X, Wang Y, Shen J, Wu WK, Liang J and Feng F. By downregulating TIAM1 expression, microRNA-329 suppresses gastric cancer invasion and growth. Oncotarget. 2015; 6:17559-17569.

83. Tang YQ, Zhang WH, Li MB and Yan L. miR-10b represses the proliferation and invasion of prostate cancer by targeting LRH1. Int J Clin Exp Pathol. 2016; 9:1424-1431.

84. Lin QS, Aihara A, Chung WH, Li Y, Chen XS, Huang ZP, Weng SF, Carlson RI, Nadolny C, Wands JR and Dong XQ. LRH1 promotes pancreatic cancer metastasis. Cancer Lett. 2014; 350:15-24.

85. Lin QS, Aihara A, Chung WH, Li Y, Huang ZP, Chen XS, Weng SF, Carlson RI, Wands JR and Dong XQ. LRH1 as a driving factor in pancreatic cancer growth. Cancer Lett. 2014; 345:85-90.

86. Gabriely G, Yi M, Narayan RS, Niers JM, Wurdinger T, Imitola J, Ligon KL, Kesari S, Esau C, Stephens RM, Tannous BA and Krichevsky AM. Human glioma growth is controlled by microRNA-10b. Cancer Res. 2011; 71:3563-3572.

87. Sionov RV, Vlahopoulos SA and Granot Z. Regulation of Bim in Health and Disease. Oncotarget. 2015; 6:23058-23134.

88. Kang J, Kim W, Lee S, Kwon D, Chun J, Son B, Kim E, Lee JM, Youn H and Youn B. TFAP2C promotes lung tumorigenesis and aggressiveness through miR-183- and miR-33a-mediated cell cycle regulation. Oncogene. 2016.

89. Yamashita H, Zheng Z, Osei Amponsa V, Raman J and DeGraff D. MP68-10 EXPRESSION OF TFAP2C IS ASSOCIATED WITH THE BASAL MOLECULAR SUBTYPE OF BLADDER CANCER AND INCREASED TUMOR AGGRESSIVENESS. The Journal of Urology. 2015; 193:e861.

90. Abbas T and Dutta A. p21 in cancer: intricate networks and multiple activities. Nat Rev Cancer. 2009; 9:400-414.

91. Xie DY, Lan LH, Huang KT, Chen L, Xu CC, Wang RR, Shi Y, Wu XY, Wang L, Liu YZ and Lu B. Association of p53/p21 expression and cigarette smoking with tumor progression and poor prognosis in non-small cell lung cancer patients. Oncol Rep. 2014; 32:2517-2526.

92. Wang X, Li Y, Qin J, Zhao H and Zhao T. [The expression of p16 and Rb proteins in non-small cell lung cancer]. Zhongguo Fei Ai Za Zhi. 2001; 4:63-65.

93. Chen JT, Chen YC, Chen CY and Wang YC. Loss of p16 and/or pRb protein expression in NSCLC. An immunohistochemical and prognostic study. Lung Cancer. 2001; 31:163-170.

94. Myong NH. Cyclin D1 overexpression, p16 loss, and pRb inactivation play a key role in pulmonary carcinogenesis and have a prognostic implication for the long-term survival in non-small cell lung carcinoma patients. Cancer Res Treat. 2008; 40:45-52.

95. Romagosa C, Simonetti S, Lopez-Vicente L, Mazo A, Lleonart ME, Castellvi J and Cajal SRY. p16(Ink4a) overexpression in cancer: a tumor suppressor gene associated with senescence and high-grade tumors. Oncogene. 2011; 30:2087-2097.

96. Ma ZH, Chen YR, Min LS, Li LQ, Huang HL, Li J, Yan Q, Song PT, Dai LC and Yao X. Augmented miR-10b expression associated with depressed expression of its target gene KLF4 involved in gastric carcinoma. Int J Clin Exp Pathol. 2015; 8:5071-5079.

97. Yu T, Chen X, Zhang W, Liu J, Avdiushko R, Napier DL, Liu AX, Neltner JM, Wang C, Cohen D and Liu C. KLF4 regulates adult lung tumor-initiating cells and represses K-Ras-mediated lung cancer. Cell Death and Differentiation. 2016; 23:207-215.

98. Chen X, Johns DC, Geiman DE, Marban E, Dang DT, Hamlin G, Sun R and Yang VW. Kruppel-like factor 4 (gut-enriched Kruppel-like factor) inhibits cell proliferation by blocking G1/S progression of the cell cycle. J Biol Chem. 2001; 276:30423-30428.

99. Wei D, Gong W, Kanai M, Schlunk C, Wang L, Yao JC, Wu TT, Huang S and Xie K. Drastic down-regulation of Kruppel-like factor 4 expression is critical in human gastric cancer development and progression. Cancer Res. 2005; 65:2746-2754.

100. Kim J, Siverly AN, Chen D, Wang M, Yuan Y, Wang Y, Lee H, Zhang J, Muller WJ, Liang H, Gan B, Yang X, Sun Y, et al. Ablation of miR-10b Suppresses Oncogene-Induced Mammary Tumorigenesis and Metastasis and Reactivates Tumor-Suppressive Pathways. Cancer Res. 2016.

101. Wang F, Yang XY, Zhao JY, Yu LW, Zhang P, Duan WY, Chong M and Gui YH. miR-10a and miR-10b target the 3'-untranslated region of TBX5 to repress its expression. Pediatr Cardiol. 2014; 35:1072-1079.

102. Yu J, Ma X, Cheung KF, Li X, Tian L, Wang S, Wu CW, Wu WK, He M, Wang M, Ng SS and Sung JJ. Epigenetic inactivation of T-box transcription factor 5, a novel tumor suppressor gene, is associated with colon cancer. Oncogene. 2010; 29:6464-6474.

103. Guo Y, Lang X, Lu Z, Wang JC, Li T, Liao Y, Jia CY, Zhao WX and Fang HQ. MiR-10b Directly Targets ZEB1 and PIK3CA to Curb Adenomyotic Epithelial Cell Invasiveness via Upregulation of E-Cadherin and Inhibition of Akt Phosphorylation. Cell Physiol Biochem. 2015; 35:2169-2180.

104. Angulo B, Suarez-Gauthier A, Lopez-Rios F, Medina PP, Conde E, Tang M, Soler G, Lopez-Encuentra A, Cigudosa JC and Sanchez-Cespedes M. Expression signatures in lung cancer reveal a profile for EGFR-mutant tumours and identify selective PIK3CA overexpression by gene amplification. J Pathol. 2008; 214:347-356.

105. Zhao Q, Zhang B, Shao Y, Chen L, Wang X, Zhang Z, Shu Y and Guo R. Correlation between the expression levels of miR-1 and PIK3CA in non-small-cell lung cancer and their relationship with clinical characteristics and prognosis. Future Oncol. 2014; 10:49-57.

106. Du L, Chen X, Cao Y, Lu L, Zhang F, Bornstein S, Li Y, Owens P, Malkoski S, Said S, Jin F, Kulesz-Martin M, Gross N, et al. Overexpression of PIK3CA in murine head and neck epithelium drives tumor invasion and metastasis through PDK1 and enhanced TGFbeta signaling. Oncogene. 2016; 35:4641-4652.

107. Chen Y, Hou Q, Yan W, Luo J, Chen D, Liu Z, He S and Ding X. PIK3CA is critical for the proliferation, invasiveness, and drug resistance of human tongue carcinoma cells. Oncol Res. 2011; 19:563-571.

108. Crowder RJ, Phommaly C, Tao Y, Hoog J, Luo J, Perou CM, Parker JS, Miller MA, Huntsman DG, Lin L, Snider J, Davies SR, Olson JA, Jr., et al. PIK3CA and PIK3CB inhibition produce synthetic lethality when combined with estrogen deprivation in estrogen receptor-positive breast cancer. Cancer Res. 2009; 69:3955-3962.

109. Roper J, Sinnamon MJ, Coffee EM, Belmont P, Keung L, Georgeon-Richard L, Wang WV, Faber AC, Yun J, Yilmaz OH, Bronson RT, Martin ES, Tsichlis PN, et al. Combination PI3K/MEK inhibition promotes tumor apoptosis and regression in PIK3CA wild-type, KRAS mutant colorectal cancer. Cancer Lett. 2014; 347:204-211.

110. Schneider C, Kässens N, Greve B, Hassan H, Schüring AN, Starzinski-Powitz A, Kiesel L, Seidler DG and Götte M. Targeting of syndecan-1 by micro-ribonucleic acid miR-10b modulates invasiveness of endometriotic cells via dysregulation of the proteolytic milieu and interleukin-6 secretion. Fertility and Sterility. 2013; 99:871-881.e871.

111. Ibrahim SA, Yip GW, Stock C, Pan JW, Neubauer C, Poeter M, Pupjalis D, Koo CY, Kelsch R, Schule R, Rescher U, Kiesel L and Gotte M. Targeting of syndecan-1 by microRNA miR-10b promotes breast cancer cell motility and invasiveness via a Rho-GTPase- and E-cadherin-dependent mechanism. Int J Cancer. 2012; 131:E884-E896.

112. Anttonen A, Heikkila P, Kajanti M, Jalkanen M and Joensuu H. High syndecan-1 expression is associated with favourable outcome in squamous cell lung carcinoma treated with radical surgery. Lung Cancer. 2001; 32:297-305.

113. Peterfia B, Fule T, Baghy K, Szabadkai K, Fullar A, Dobos K, Zong F, Dobra K, Hollosi P, Jeney A, Paku S and Kovalszky I. Syndecan-1 Enhances Proliferation, Migration and Metastasis of HT-1080 Cells in Cooperation with Syndecan-2. PLoS One. 2012; 7.

114. Maeda T, Alexander CM and Friedl A. Induction of syndecan-1 expression in stromal fibroblasts promotes proliferation of human breast cancer cells. Cancer Research. 2004; 64:612-621.

115. Purushothaman A, Uyama T, Kobayashi F, Yamada S, Sugahara K, Rapraeger AC and Sanderson RD. Heparanase-enhanced shedding of syndecan-1 by myeloma cells promotes endothelial invasion and angiogenesis. Blood. 2010; 115:2449-2457.

116. Oh JH, Kim JH, Ahn HJ, Yoon JH, Yoo SC, Choi DS, Lee IS, Ryu HS and Min CK. Syndecan-1 enhances the endometrial cancer invasion by modulating matrix metalloproteinase-9 expression through nuclear factor kappaB. Gynecol Oncol. 2009; 114:509-515.

117. Khotskaya YB, Dai YM, Ritchie JP, MacLeod V, Yang Y, Zinn K and Sanderson RD. Syndecan-1 Is Required for Robust Growth, Vascularization, and Metastasis of Myeloma Tumors in Vivo. J Biol Chem. 2009; 284:26085-26095.

118. Zong F, Fthenou E, Castro J, Peterfia B, Kovalszky I, Szilak L, Tzanakakis G and Dobra K. Effect of syndecan-1 overexpression on mesenchymal tumour cell proliferation with focus on different functional domains. Cell Prolif. 2010; 43:29-40.

119. Ishikawa T and Kramer RH. Sdc1 negatively modulates carcinoma cell motility and invasion. Exp Cell Res. 2010; 316:951-965.

120. Beauvais DM, Jung O, Yang Y, Sanderson RD and Rapraeger AC. Syndecan-1 (CD138) Suppresses Apoptosis in Multiple Myeloma by Activating IGF1 Receptor: Prevention by SynstatinIGF1R Inhibits Tumor Growth. Cancer Res. 2016; 76:4981-4993.

121. Wu YH, Yang CY, Chien WL, Lin KI and Lai MZ. Removal of syndecan-1 promotes TRAIL-induced apoptosis in myeloma cells. J Immunol. 2012; 188:2914-2921.

122. Liebersbach BF and Sanderson RD. Expression of syndecan-1 inhibits cell invasion into type I collagen. J Biol Chem. 1994; 269:20013-20019.

123. Chen Y, Liu W, Chao T, Zhang Y, Yan X, Gong Y, Qiang B, Yuan J, Sun M and Peng X. MicroRNA-21 down-regulates the expression of tumor suppressor PDCD4 in human glioblastoma cell T98G. Cancer Lett. 2008; 272:197-205.

124. Asangani IA, Rasheed SA, Nikolova DA, Leupold JH, Colburn NH, Post S and Allgayer H. MicroRNA-21 (miR-21) post-transcriptionally downregulates tumor suppressor Pdcd4 and stimulates invasion, intravasation and metastasis in colorectal cancer. Oncogene. 2008; 27:2128-2136.

125. Frankel LB, Christoffersen NR, Jacobsen A, Lindow M, Krogh A and Lund AH. Programmed cell death 4 (PDCD4) is an important functional target of the microRNA miR-21 in breast cancer cells. J Biol Chem. 2008; 283:1026-1033.

126. Selaru FM, Olaru AV, Kan T, David S, Cheng Y, Mori Y, Yang J, Paun B, Jin Z, Agarwal R, Hamilton JP, Abraham J, Georgiades C, et al. MicroRNA-21 is overexpressed in human cholangiocarcinoma and regulates programmed cell death 4 and tissue inhibitor of metalloproteinase 3. Hepatology. 2009; 49:1595-1601.

127. Chen Y, Knosel T, Kristiansen G, Pietas A, Garber ME, Matsuhashi S, Ozaki I and Petersen I. Loss of PDCD4 expression in human lung cancer correlates with tumour progression and prognosis. J Pathol. 2003; 200:640-646.

128. Zhen Y, Li DM, Li W, Yao WM, Wu AB, Huang J, Gu HL, Huang YJ, Wang YJ, Wu J, Chen M, Wu D, Lyu QC, et al. Reduced PDCD4 Expression Promotes Cell Growth Through PI3K/Akt Signaling in Non-Small Cell Lung Cancer. Oncol Res. 2015; 23:61-68.

129. Wei ZT, Zhang X, Wang XY, Gao F, Zhou CJ, Zhu FL, Wang Q, Gao Q, Ma CH, Sun WS, Fu QZ, Chen YH and Zhang LN. PDCD4 inhibits the malignant phenotype of ovarian cancer cells. Cancer Sci. 2009; 100:1408-1413.

130. Yang HS, Matthews CP, Clair T, Wang Q, Baker AR, Li CC, Tan TH and Colburn NH. Tumorigenesis suppressor Pdcd4 down-regulates mitogen-activated protein kinase kinase kinase kinase 1 expression to suppress colon carcinoma cell invasion. Mol Cell Biol. 2006; 26:1297-1306.

131. Wang N, Zhang CQ, He JH, Duan XF, Wang YY, Ji X, Zang WQ, Li M, Ma YY, Wang T and Zhao GQ. miR-21 Down-Regulation Suppresses Cell Growth, Invasion and Induces Cell Apoptosis by Targeting FASL, TIMP3, and RECK Genes in Esophageal Carcinoma. Dig Dis Sci. 2013; 58:1863-1870.

132. Hu JZ, Ni SF, Cao Y, Zhang T, Wu TD, Yin XZ, Lang Y and Lu HB. The Angiogenic Effect of microRNA-21 Targeting TIMP3 through the Regulation of MMP2 and MMP9. PLoS One. 2016; 11.

133. Kettunen E, Anttila S, Seppanen JK, Karjalainen A, Edgren H, Lindstrom I, Salovaara R, Nissen AM, Salo J, Mattson K, Hollmen J, Knuutila S and Wikman H. Differentially expressed genes in nonsmall cell lung cancer: expression profiling of cancer-related genes in squamous cell lung cancer. Cancer Genet Cytogenet. 2004; 149:98-106.

134. Wu MS, Tu T, Huang YC and Cao Y. Suppression subtractive hybridization identified differentially expressed genes in lung adenocarcinoma: ERGIC3 as a novel lung cancer-related gene. BMC Cancer. 2013; 13.

135. Mashkova TD, Oparina N, Zinov'eva OL, Kropotova ES, Dubovaia VI, Poltaraus AB, Fridman MV, Kopantsev EP, Vinogradova TV, Zinov'eva MV, Laktionov KK, Kasymova OT, Zborovskaia IB, et al. [Transcription TIMP3, DAPk1 and AKR1B10 genes in squamous cell lung cancer]. Mol Biol (Mosk). 2006; 40:1047-1054.

136. Kong LZ, Zhang P, Li W, Yang Y, Tian Y, Wang XJ, Chen SJ, Yang YX, Huang TH, Zhao T, Tang L, Su B, Li F, et al. KDM1A promotes tumor cell invasion by silencing TIMP3 in non-small cell lung cancer cells. Oncotarget. 2016; 7:27959-27974.

137. Das AM, Seynhaeve AL, Rens JA, Vermeulen CE, Koning GA, Eggermont AM and Ten Hagen TL. Differential TIMP3 expression affects tumor progression and angiogenesis in melanomas through regulation of directionally persistent endothelial cell migration. Angiogenesis. 2014; 17:163-177.

138. Han XG, Li Y, Mo HM, Li K, Lin D, Zhao CQ, Zhao J and Tang TT. TIMP3 regulates osteosarcoma cell migration, invasion, and chemotherapeutic resistances. Tumor Biol. 2016; 37:8857-8867.

139. Qi JH and Anand-Apte B. Tissue inhibitor of metalloproteinase-3 (TIMP3) promotes endothelial apoptosis via a caspase-independent mechanism. Apoptosis. 2015; 20:523-534.

140. Zhu S, Si ML, Wu H and Mo YY. MicroRNA-21 targets the tumor suppressor gene tropomyosin 1 (TPM1). J Biol Chem. 2007; 282:14328-14336.

141. Zhu S, Wu H, Wu F, Nie D, Sheng S and Mo YY. MicroRNA-21 targets tumor suppressor genes in invasion and metastasis. Cell Res. 2008; 18:350-359.

142. Du HQ, Wang Y, Jiang Y, Wang CH, Zhou T, Liu HY and Xiao H. Silencing of the TPM1 gene induces radioresistance of glioma U251 cells. Oncol Rep. 2015; 33:2807-2814.

143. Han L, Yue X, Zhou X, Lan FM, You G, Zhang W, Zhang KL, Zhang CZ, Cheng JQ, Yu SZ, Pu PY, Jiang T and Kang CS. MicroRNA-21 expression is regulated by beta-catenin/STAT3 pathway and promotes glioma cell invasion by direct targeting RECK. CNS Neurosci Ther. 2012; 18:573-583.

144. Chang HC, Cho CY and Hung WC. Downregulation of RECK by promoter methylation correlates with lymph node metastasis in non-small cell lung cancer. Cancer Sci. 2007; 98:169-173.

145. Oh J, Takahashi R, Kondo S, Mizoguchi A, Adachi E, Sasahara RM, Nishimura S, Imamura Y, Kitayama H, Alexander DB, Ide C, Horan TP, Arakawa T, et al. The membrane-anchored MMP inhibitor RECK is a key regulator of extracellular matrix integrity and angiogenesis. Cell. 2001; 107:789-800.

146. Takenaka K, Ishikawa S, Kawano Y, Yanagihara K, Miyahara R, Otake Y, Morioka Y, Takahashi C, Noda M, Wada H and Tanaka F. Expression of a novel matrix metalloproteinase regulator, RECK, and its clinical significance in resected non-small cell lung cancer. Eur J Cancer. 2004; 40:1617-1623.

147. Zhong Z, Dong Z, Yang L and Gong Z. miR-21 induces cell cycle at S phase and modulates cell proliferation by down-regulating hMSH2 in lung cancer. J Cancer Res Clin Oncol. 2012; 138:1781-1788.

148. Zhang YX, Yue Z, Wang PY, Li YJ, Xin JX, Pang M, Zheng QY and Xie SY. Cisplatin upregulates MSH2 expression by reducing miR-21 to inhibit A549 cell growth. Biomed Pharmacother. 2013; 67:97-102.

149. Valeri N, Gasparini P, Braconi C, Paone A, Lovat F, Fabbri M, Sumani KM, Alder H, Amadori D, Patel T, Nuovo GJ, Fishel R and Croce CM. MicroRNA-21 induces resistance to 5-fluorouracil by down-regulating human DNA MutS homolog 2 (hMSH2). Proc Natl Acad Sci U S A. 2010; 107:21098-21103.

150. Xinarianos G, Liloglou T, Prime W, Maloney P, Callaghan J, Fielding P, Gosney JR and Field JK. hMLH1 and hMSH2 expression correlates with allelic imbalance on chromosome 3p in non-small cell lung carcinomas. Cancer Res. 2000; 60:4216-4221.

151. Kanellis G, Chatzistamou I, Koutselini H, Politi E, Gouliamos A, Vlahos L and Koutselinis A. Expression of DNA mismatch repair gene MSH2 in cytological material from lung cancer patients. Diagn Cytopathol. 2006; 34:463-466.

152. Edelbrock MA, Kaliyaperumal S and Williams KJ. Structural, molecular and cellular functions of MSH2 and MSH6 during DNA mismatch repair, damage signaling and other noncanonical activities. Mutat Res-Fundam Mol Mech Mutagen. 2013; 743:53-66.

153. Seifert M and Reichrath J. The role of the human DNA mismatch repair gene hMSH2 in DNA repair, cell cycle control and apoptosis: implications for pathogenesis, progression and therapy of cancer. J Mol Histol. 2006; 37:301-307.

154. Fishel R, Lescoe MK, Rao MR, Copeland NG, Jenkins NA, Garber J, Kane M and Kolodner R. The human mutator gene homolog MSH2 and its association with hereditary nonpolyposis colon cancer. Cell. 1993; 75:1027-1038.

155. Wang P, Zou F, Zhang X, Li H, Dulak A, Tomko RJ, Jr., Lazo JS, Wang Z, Zhang L and Yu J. microRNA-21 negatively regulates Cdc25A and cell cycle progression in colon cancer cells. Cancer Res. 2009; 69:8157-8165.

156. Wu W, Fan Y-H, Kemp BL, Walsh G and Mao L. Overexpression of cdc25A and cdc25B is frequent in primary non-small cell lung cancer but is not associated with overexpression of c-myc. Cancer Research. 1998; 58:4082-4085.

157. Hoffmann I, Draetta G and Karsenti E. Activation of the phosphatase activity of human cdc25A by a cdk2-cyclin E dependent phosphorylation at the G1/S transition. EMBO J. 1994; 13:4302-4310.

158. Blomberg I and Hoffmann I. Ectopic expression of Cdc25A accelerates the G(1)/S transition and leads to premature activation of cyclin E- and cyclin A-dependent kinases. Mol Cell Biol. 1999; 19:6183-6194.

159. Sayed D, He M, Hong C, Gao S, Rane S, Yang Z and Abdellatif M. MicroRNA-21 is a downstream effector of AKT that mediates its antiapoptotic effects via suppression of Fas ligand. J Biol Chem. 2010; 285:20281-20290.

160. Wu MF, Yang J, Xiang T, Shi YY and Liu LJ. miR-21 targets Fas ligand-mediated apoptosis in breast cancer cell line MCF-7. J Huazhong Univ Sci Tech-Med. 2014; 34:190-194.

161. Wang P, Zhuang LP, Zhang J, Fan J, Luo JM, Chen H, Wang K, Liu LM, Chen Z and Meng ZQ. The serum miR-21 level serves as a predictor for the chemosensitivity of advanced pancreatic cancer, and miR-21 expression confers chemoresistance by targeting FasL. Mol Oncol. 2013; 7:334-345.

162. Badillo-Almaraz I, Badillo-Salas C, Villalobos R, Avalos-Diaz E and Herrera-Esparza R. Defective expression of FasL and Bax in human lung cancer. Clin Exper Med. 2003; 3:106-112.

163. Ehrenschwender M and Wajant H. The role of FasL and Fas in health and disease. Adv Exp Med Biol. 2009; 647:64-93.

164. Liu M, Wu H, Liu T, Li Y, Wang F, Wan H, Li X and Tang H. Regulation of the cell cycle gene, BTG2, by miR-21 in human laryngeal carcinoma. Cell Res. 2009; 19:828-837.

165. Mao B, Xiao H, Zhang Z, Wang D and Wang G. MicroRNA21 regulates the expression of BTG2 in HepG2 liver cancer cells. Mol Med Rep. 2015; 12:4917-4924.

166. Zhang L, Huang H, Wu K, Wang M and Wu B. Impact of BTG2 expression on proliferation and invasion of gastric cancer cells in vitro. Molecular Biology Reports. 2010; 37:2579-2586.

167. Zhang Y-j, Wei L, Liu M, Li J, Zheng Y-q, Gao Y and Li X-r. BTG2 inhibits the proliferation, invasion, and apoptosis of MDA-MB-231 triple-negative breast cancer cells. Tumor Biol. 2013; 34:1605-1613.

168. Wagener N, Bulkescher J, Macher-Goeppinger S, Karapanagiotou-Schenkel I, Hatiboglu G, Abdel-Rahim M, Abol-Enein H, Ghoneim MA, Bastian PJ, Muller SC, Haferkamp A, Hohenfellner M, Hoppe-Seyler F, et al. Endogenous BTG2 expression stimulates migration of bladder cancer cells and correlates with poor clinical prognosis for bladder cancer patients. Br J Cancer. 2013; 108:973-982.
